# Artificial intelligence in ovarian cancer prevention and control: a brief review of detection, treatment, and equity (2021–2026)

**DOI:** 10.3389/fonc.2026.1915221

**Published:** 2026-09-03

**Authors:** Zhenyu Huang, Boyang Wei, Ying Shen

**Affiliations:** 1 School of Humanities and Social Sciences, Harbin Engineering University, Harbin, China; 2 Department of Obstetrics and Gynecology, Heilongjiang University of Chinese Medicine, Harbin, China

**Keywords:** artificial intelligence, early detection, health equity, ovarian cancer, precision oncology, treatment response

## Abstract

Ovarian cancer (OC) remains a leading cause of gynecologic cancer mortality worldwide because early symptoms are often vague, effective population-level screening strategies are lacking, and outcomes are shaped by biological heterogeneity and unequal access to diagnostic and molecular testing resources. This Mini Review summarizes advances from 2021 to 2026 in artificial intelligence (AI) applications across the OC care continuum, focusing on early detection, treatment decision-making, prognostic assessment, clinical implementation, and health equity. This review maps AI applications against current clinical limitations, established benchmark tools, translational readiness, and equity-related implementation gaps. In detection, AI models integrate multimarker blood panels, routine laboratory indicators, ultrasound imaging, and cell-free DNA (cfDNA)-derived molecular signals to improve risk stratification beyond traditional biomarkers such as CA125, although prospective validation remains limited. In treatment and prognosis, pathology-based, radiomics-based, omics-based, and multimodal AI models show potential for predicting homologous recombination deficiency (HRD), platinum response, recurrence risk, and survival outcomes, but most remain retrospective or externally validated rather than clinically deployed. Blood/laboratory-based and ultrasound-assisted AI appear closest to workflow-based evaluation, whereas cfDNA-based screening, population-level AI detection, and multimodal prognostic models remain preliminary. Future efforts should prioritize prospective multicenter studies, benchmarked evaluation, transparent reporting, cost-effectiveness analysis, workflow integration, and globally representative datasets. Ultimately, AI should be judged by its ability to improve timely, affordable, and equitable OC care.

## 1 Introduction

Ovarian cancer (OC) remains a lethal gynecologic malignancy, with 324,000 cases and 207,000 deaths according to GLOBOCAN 2022 (1). Its poor prognosis is driven by delayed diagnosis and stage-dependent survival differences: five-year survival exceeds 90% in stage I disease but declines to 25%–30% in advanced stages (2). About 70% of patients are diagnosed late, reflecting absent effective population screening and limitations in diagnostic pathways (2, 3). Clinical evaluation relies on pelvic examination, transvaginal ultrasound, cancer antigen 125 (CA125), human epididymis protein 4 (HE4), the Risk of Ovarian Malignancy Algorithm (ROMA), the Risk of Malignancy Index (RMI), computed tomography (CT), and histopathology. These tools are limited by insufficient early-stage sensitivity, false positives, operator dependence, and restricted access to molecular testing. OC is also biologically heterogeneous. High-grade serous ovarian cancer (HGSOC) is characterized by chromosomal instability, near-universal TP53 mutation, and frequent homologous recombination deficiency (HRD) (3, 4). Prognosis and treatment selection are shaped by International Federation of Gynecology and Obstetrics (FIGO) stage, histology, BRCA/HRD status, platinum sensitivity, and eligibility for poly(ADP-ribose) polymerase (PARP) inhibitors, underscoring the need for tools that support early detection, molecular stratification, and individualized care (3–5).

Artificial intelligence (AI), including machine learning (ML) and deep learning (DL), has emerged as a potential approach to address these gaps rather than merely provide high-performance classification (6). In early detection and triage, AI models integrate multimarker blood tests, routine laboratory data, and cell-free DNA (cfDNA) signals to improve risk stratification beyond CA125. For example, the third-generation Multivariate Index Assay (MIA3G), implemented through a deep neural network, combines serum proteins with clinical variables and has shown promising performance in low-prevalence settings (7), while a Chinese multicenter study reported better diagnostic performance than CA125 alone, particularly in early-stage disease (8). Imaging-based AI should be interpreted alongside established frameworks such as the Ovarian-Adnexal Reporting and Data System (O-RADS) and International Ovarian Tumor Analysis (IOTA) models, which guide ultrasound risk assessment. cfDNA-based AI may support noninvasive molecular detection but remains less mature than diagnostic pathways (9, 10). Across these domains, the key question is whether AI provides incremental value beyond existing clinical tools. For treatment response and prognosis, deep learning using hematoxylin and eosin (H&E)-stained slides can predict platinum response and HRD status, but should complement, rather than replace, genomic testing (11, 12). Multimodal AI integrating pathology, imaging, and clinical data may enhance prognostic assessment beyond conventional factors such as FIGO stage, residual disease, BRCA/HRD status, platinum sensitivity, and CA-125 elimination rate constant K (KELIM) (13, 14).

**Figure 1:**
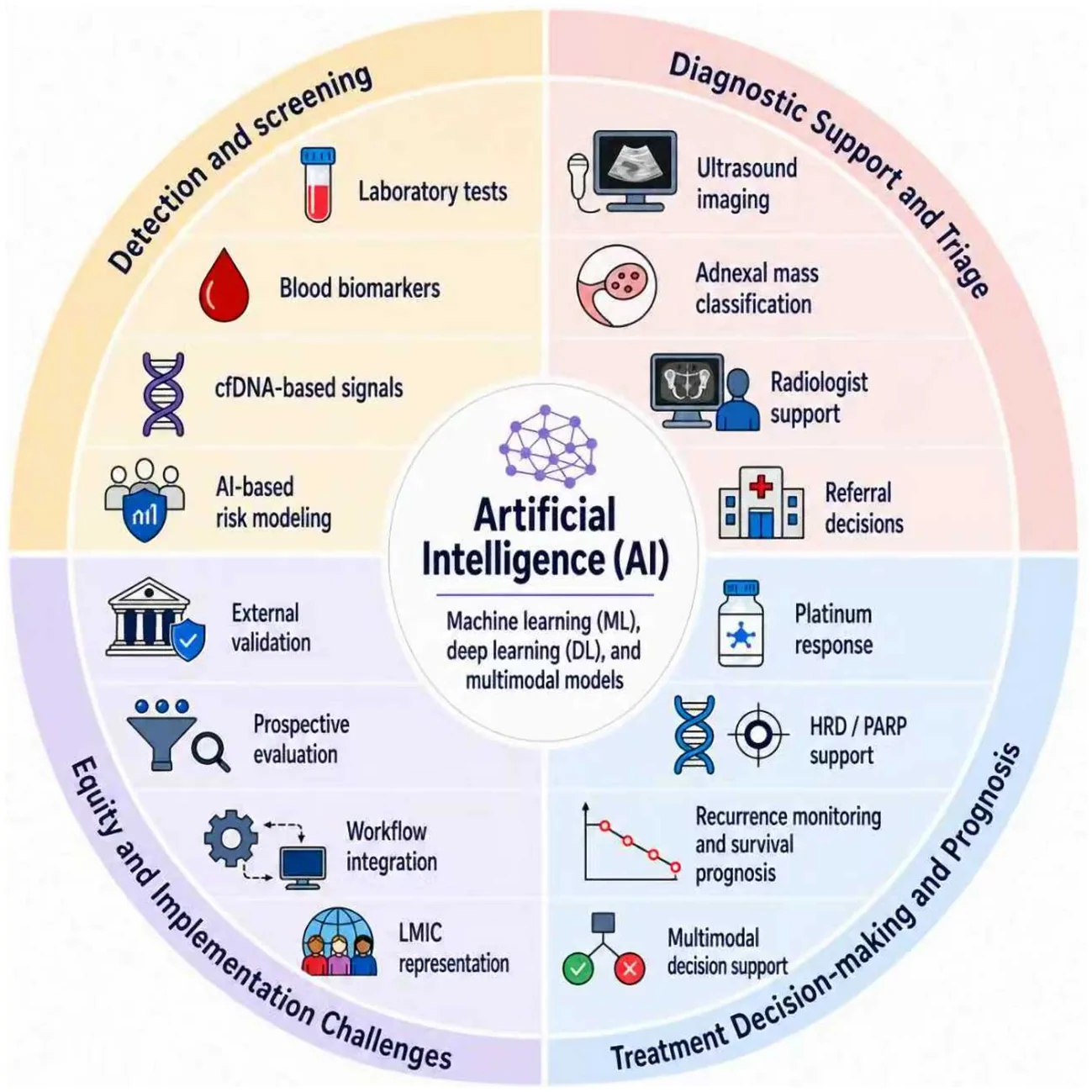
AI-driven framework for ovarian cancer management. This figure summarizes a circular framework of artificial intelligence (AI) applications across ovarian cancer care, integrating multimodal data for clinical decision support. AI may support early detection and risk stratification, diagnostic support and triage, treatment decision-making and prognosis prediction, and addresses equity and implementation challenges including validation, workflow integration, and LMIC representation. The model represents a continuous AI-assisted clinical decision pipeline from detection to implementation.

Although recent reviews have summarized AI applications in OC (6, 15, 16), fewer have examined whether these models address clinical limitations, are benchmarked against established tools, or demonstrate translational readiness. This Mini Review maps AI applications against clinical gaps and comparators, including CA125, HE4, ROMA, RMI, O-RADS, IOTA models, genomic HRD testing, FIGO stage, residual disease, and KELIM. By organizing evidence around clinical limitations and AI-added value, this review aims to provide a clinically relevant synthesis rather than a descriptive catalogue. The analytical framework is summarized in **Figure 1**, illustrating an AI-driven clinical decision pipeline that maps clinical limitations, benchmark comparators, AI-added value, and translational gaps from early detection to deployment.

## 2 AI in ovarian cancer detection and screening

AI is increasingly applied to ovarian cancer detection because conventional approaches remain insufficient for early identification. Current evaluation relies on serum biomarkers, transvaginal ultrasound, cross-sectional imaging, and histopathological confirmation, but these methods remain limited when used alone (3). Here, detection refers to diagnostic support, lesion characterization, and risk stratification, whereas screening refers to population-level identification, which remains largely unvalidated for AI in ovarian cancer. Therefore, AI models should be assessed against established comparators, false-positive harms, positive predictive value, workflow feasibility, and discrimination metrics.

### 2.1 Blood-based and laboratory-based AI models

Blood- and laboratory-based AI models should be interpreted against established triage tools such as CA125, HE4, ROMA, RMI, and OVA1/MIA assays. Conventional biomarkers often show limited early-stage sensitivity and false positives in benign gynecological conditions. AI models aim to address these limitations by integrating weak biological signals and laboratory parameters. Their clinical value depends on whether they improve referral accuracy, early-stage risk stratification, and false-positive control beyond existing approaches.

Multimarker integration can improve diagnostic performance over single biomarkers. The third-generation Multivariate Index Assay (MIA3G) deep neural network combined seven serum protein biomarkers with age and menopausal status, showing promising diagnostic performance in simulated low-prevalence settings (7). The MCF model showed better diagnostic performance than CA125 alone, particularly in early-stage disease (8). Another model using demographic variables and 28 blood indicators reported favorable performance (17). A meta-analysis reported pooled sensitivity of 85%, specificity of 91%, and area under the receiver operating characteristic curve (AUC) of 0.95 for AI-derived blood biomarkers (18). However, prospective validation remains scarce, and retrospective case-control studies are vulnerable to spectrum bias. Direct comparisons with ROMA, RMI, and OVA1/MIA assays remain limited, restricting conclusions about incremental clinical value. False-positive control is critical in low-prevalence settings, where false positives may lead to unnecessary tests, invasive procedures, surgery, and psychological distress. Thus, blood-based AI is best viewed as complementary diagnostic support rather than standalone screening.

### 2.2 Imaging-based AI models

Imaging is central to ovarian cancer evaluation, particularly for adnexal mass assessment. Although AI may enhance ultrasound, CT, and MRI interpretation by identifying subtle patterns not consistently recognized by clinicians (3, 6), most evidence focuses on ultrasound. O-RADS, IOTA Simple Rules, and the IOTA ADNEX model already guide ultrasound-based risk stratification; therefore, imaging AI should be evaluated for added value beyond these systems, especially for indeterminate masses, non-expert settings, and multicenter workflows.

Deep learning and radiomics may refine adnexal lesion characterization. An ultrasound-based deep learning pipeline was validated in a multicenter retrospective cohort (19). A radiomics model differentiated benign, borderline, and malignant tumors, achieving micro- and macro-average AUCs of 0.90 and 0.84 (20). Classification performance improved when ultrasound data were combined with menopausal status and serum biomarkers (21), and Clinical-OMTA increased external validation accuracy from 72.3% to 88.0% after integrating ultrasound images, age, and CA125 (22). These findings suggest that imaging AI may improve diagnostic consistency, but many studies still lack head-to-head benchmarking against O-RADS or IOTA-based models. Future studies should test whether AI can refine risk stratification of O-RADS indeterminate lesions or match IOTA ADNEX performance in community hospitals. Scanner variability and operator-dependent acquisition remain key barriers.

### 2.3 cfDNA-based molecular detection and AI integration

Compared with established triage tools such as CA125, HE4, transvaginal ultrasound, and multimarker assays, cfDNA-based AI remains at an earlier translational stage. Its value for population screening cannot be inferred from case-control performance alone and requires prospective validation in low-prevalence settings.

Cell-free DNA (cfDNA) approaches extend detection toward molecular signals.Plasma cfDNA methylation using cell-free methylated DNA immunoprecipitation and sequencing (cfMeDIP-seq) has distinguished ovarian cancer from benign lesions and healthy controls (23).However, late-stage-derived biomarkers may underperform in early disease. The WID-cfOC score improves sensitivity while maintaining specificity when combined with CA125 (24). DELFI-Pro integrates cfDNA fragmentomics, chromosomal alterations, CA125, and HE4, outperforming individual biomarkers in differentiating malignant from benign adnexal masses (25). However, variable pre-diagnostic performance, sequencing costs, bioinformatics infrastructure, and lack of pre-analytical standardization restrict general population use.

Overall, blood models support early risk stratification, imaging tools refine lesion characterization, and cfDNA approaches offer noninvasive molecular insights. Translation requires clinical benchmarking against standard pathways such as ROMA, RMI, O-RADS, and IOTA ADNEX, together with false-positive control, cost-effectiveness assessment, and prospective real-world trials. To place these studies within a broader evidence base, **Table 1** summarizes representative empirical AI applications across detection, treatment, and prognosis, while also highlighting their clinical comparators, translational value, and remaining gaps.

**Table 1:** Representative empirical AI studies, clinical comparators, and translational readiness in ovarian cancer.

Study	Clinical application	Existing benchmark/comparator	Data and AI approach	Key finding	Readiness/limitation
Detection and screening					
Reilly et al., 2022/MIA3G (7)	Ovarian cancer risk assessment	CA125, HE4, ROMA, RMI, OVA1/MIA assays; low-prevalence triage settings	Serum biomarkers + DNN	MIA3G used seven protein biomarkers, age, and menopausal status; sensitivity 89.8%, specificity 84.0%, and NPV 99.4% in analytical validation	TRL 2; analytical validation only; clinical utility, false-positive control, and population-level screening value still need further evaluation
Cai et al., 2024/MCF (8)	Detection and diagnosis	CA125, HE4, conventional laboratory-based diagnostic pathways	Laboratory data + ML ensemble	MCF used 51 laboratory tests and age, achieving AUC 0.949 internally and 0.882/0.884 in two external validation sets; it showed better diagnostic performance than CA125 and HE4, especially in early-stage disease	TRL 3; multicenter retrospective validation available, but no prospective workflow evaluation
Dai et al., 2024/OvaMTA (19)	Ovarian mass diagnosis	O-RADS, IOTA Simple Rules, IOTA ADNEX model, expert ultrasound assessment	Ultrasound + DL	OvaMTA achieved AUC 0.941 in image-based internal and external testing and AUC 0.911 in video-based external testing; performance was comparable to senior radiologists	TRL 3; multicenter retrospective validation available, but no prospective clinical evaluation
Wu et al., 2026/Clinical-OMTA (22)	Adnexal mass classification	O-RADS, IOTA ADNEX model, conventional ultrasound-based triage	Ultrasound + age + CA125	Clinical-OMTA classified benign, borderline, and malignant adnexal masses; external image AUCs were 0.950, 0.870, and 0.930, respectively	TRL 3; external testing available, but implementation and real-world workflow evidence remain limited
Representative cfDNA-based models (23–25)	cfDNA-based molecular detection	CA125, HE4, transvaginal ultrasound, multimarker assays	cfDNA methylation/fragmentomics + ML	cfDNA methylation and fragmentomic models distinguished OC from healthy controls or benign masses; DELFI-Pro combined cfDNA fragmentome features, chromosomal alterations, CA125, and HE4 and achieved AUC 0.96 with high specificity	TRL 3; sequencing cost, pre-analytical standardization, and prospective screening validation remain major barriers
Treatment decision-making and prognosis					
Ahn et al., 2024/PathoRiCH (11)	Platinum-response prediction	BRCA/HRD status, platinum-free interval, conventional platinum-sensitivity assessment	H&E pathology slides + DL	PathoRiCH was trained on 394 HGSOC cases and validated on two external cohorts of 284 and 136 cases; it stratified favorable and poor platinum-response groups with different platinum-free intervals	TRL 3; retrospective design with external validation; prospective validation and workflow integration are still needed
Bergstrom et al., 2024/DeepHRD (12)	HRD prediction and treatment decision support	BRCA/HRD genomic testing, platinum sensitivity, PARP inhibitor eligibility	H&E pathology slides + DL	DeepHRD predicted HRD from routine H&E slides; reported AUC was 0.81 for HRD prediction, and DeepHRD-predicted HRD was associated with better OS in HGSOC after platinum therapy	TRL 4; cross-cohort validation available, but it should support rather than replace validated genomic HRD testing
Gao et al., 2025/GPPS (27)	Platinum response and prognosis	Platinum-free interval, BRCA/HRD status, KELIM, clinicopathological prognostic factors	Bulk RNA-seq + scRNA-seq + RF	Five-gene GPPS model achieved AUC 0.993 in the test cohort and 0.989 in an independent validation cohort for platinum-response prediction	TRL 3; retrospective omics-based model; prospective clinical utility and cross-platform reproducibility remain untested
Zhou et al., 2026/OvcaSurvivor (13)	Survival prognosis	FIGO stage, residual disease, BRCA/HRD status, KELIM, clinical prognostic models	Pathology + ultrasound + clinical variables; multimodal DL	OvcaSurvivor integrated WSI, ultrasound, and clinical data from 543 patients; C-index was 0.81 internally and 0.76/0.70 in two external cohorts	TRL 3; external validation available, but prospective clinical utility and direct comparison with established prognostic models remain limited
Leng et al., 2025 (29)	PFS prediction	FIGO stage, residual disease, conventional CT assessment, clinical prognostic models	CT radiomics + clinical variables	CT-based radiomics nomogram predicted PFS; C-index was 0.78 in training and 0.73 in test cohorts; 1-, 3-, and 5-year AUCs were 0.850, 0.828, and 0.845	TRL 3; small retrospective cohort with limited external and prospective validation
Tang et al., 2026/multimodal pathology-clinical model (30)	Platinum response and recurrence risk	Platinum-free interval, BRCA/HRD status, recurrence-risk assessment, multidisciplinary clinical assessment	Primary/metastatic pathology images + clinical variables; multimodal DL	A multimodal pathology-clinical model integrated WSIs and clinicopathological variables; platinum-response AUC improved to 0.914 in the internal test set, and recurrence-risk C-index increased from 0.801 to 0.838 after multimodal enhancement	TRL 3; retrospective multimodal design; prospective validation, workflow testing, and head-to-head comparison with clinical assessment remain needed
Ma et al., 2025 (31)	Treatment decision support	Multidisciplinary surgical assessment, imaging-based resectability assessment, primary debulking surgery versus neoadjuvant chemotherapy decision-making	Diagnostic laparoscopy images + DL	Pretreatment laparoscopy-based DL framework predicted treatment-related outcomes in HGSOC; AUCs were above 0.80 in cross-validation	TRL 3; small cohort and no external validation; clinical value for surgical decision-making remains unproven

This table summarizes representative empirical studies of artificial intelligence (AI) in ovarian cancer (OC), covering detection, diagnosis, treatment response, and prognostic prediction. The added comparator column maps each AI application to relevant existing clinical tools, standards, or decision pathways. Technology readiness levels (TRLs) were adapted to reflect model maturity from analytical validation and retrospective development to external validation and limited clinical workflow evaluation. Overall, AI applications remain primarily at the retrospective or externally validated stage, with limited prospective testing, head-to-head benchmarking, or real-world clinical deployment.

## 3 AI in treatment decision-making and prognosis

AI is increasingly used in ovarian cancer (OC) treatment planning and prognosis because outcomes vary within similar stages or histologies. Conventional decisions rely on FIGO stage, residual disease, BRCA1/2 and HRD status, platinum sensitivity, platinum-free interval, KELIM, and clinician judgment. These factors remain essential but incompletely capture tumor heterogeneity and individual response. Thus, available treatment- and prognosis-oriented AI studies should be interpreted in relation to established comparators, including FIGO stage, residual disease, BRCA/HRD genomic testing, platinum-free interval, KELIM, and conventional prognostic models.

### 3.1 Pathology-based and omics-based AI for treatment response prediction

Pathology-based AI is clinically relevant because hematoxylin and eosin (H&E)-stained slides contain morphological information linked to molecular alterations and therapeutic response. Genomic HRD testing remains the reference standard, but cost, turnaround time, tissue availability, platform differences, and uneven access may limit timely use. PathoRiCH was developed to predict platinum chemotherapy response in high-grade serous ovarian cancer (HGSOC) and improved risk stratification when combined with BRCA1/2/HRD status (11). DeepHRD suggested that HRD-related patterns can be predicted from histopathology images, which matters because HRD is associated with platinum and PARP inhibitor sensitivity (12). These models may support response stratification and prioritize molecular testing when genomic testing is delayed or unavailable.

However, pathology-based AI should be framed as a complementary stratification tool rather than a substitute for validated BRCA/HRD testing. Its value depends on whether it can inform platinum- or PARP inhibitor-related decisions and remain reproducible across pathology workflows. Omics-based AI adds predictive information. A systematic review of 39 machine-learning models reported pooled concordance indices (C-indices) of 0.806 and 0.831 for platinum response prediction, although heterogeneity and bias remained substantial (26). A five-gene random forest model integrating bulk and single-cell RNA sequencing data showed promising validation performance (27). Most omics models remain retrospective and require standardization, reproducibility, and prospective testing.

### 3.2 AI-assisted prognosis and survival prediction

Prognostic AI models should be benchmarked against FIGO stage, residual disease, tumor grade, BRCA/HRD status, platinum sensitivity, platinum-free interval, KELIM, and existing prognostic models. AI expands prognostic modeling by extracting features from tissue, blood data, and images. Graph-based deep learning captures spatial relationships associated with survival and treatment response (14). A machine-learning blood risk score using 33 preoperative blood features and age achieved a C-index of 0.711 and improved model discrimination when combined with FIGO stage (28). CT-based radiomics nomograms achieved C-indices of 0.78 and 0.73 for progression-free survival (PFS) prediction (29). Future work should distinguish overall survival (OS), PFS, recurrence risk, and treatment-specific response. Clinical relevance should be judged by whether AI improves treatment allocation or recurrence monitoring beyond discrimination alone.

### 3.3 Multimodal and laparoscopy-based ai for individualized decision-making

Because treatment decisions depend on tumor biology, disease burden, surgical feasibility, and patient factors, multimodal AI reflects clinical complexity better than single-modality models. The benchmark is not only algorithmic performance but also existing clinical decision-making for surgical planning, treatment selection, and recurrence-risk assessment. OvcaSurvivor integrated pathology slides, ultrasound images, and clinical variables, achieving C-indices up to 0.81 and showing better performance than single-modality approaches (13). Another multimodal deep learning model combined pathology images with clinicopathological variables to predict platinum response and recurrence risk (30). Pretreatment laparoscopy-based deep learning achieved AUCs above 0.80 (31).

However, whether such models outperform established clinical prognostic models or multidisciplinary clinical assessment remains unclear. The applicability of laparoscopy-based AI is also limited by small sample size and lack of external validation. Future studies should test whether these tools improve decisions such as primary debulking surgery versus neoadjuvant chemotherapy in multidisciplinary workflows.

In conclusion, AI for OC treatment decision-making and prognosis is moving toward multimodal systems. Future studies should prespecify clinical benchmarks and prospectively evaluate calibration, clinical utility, workflow integration, and patient outcomes. Direct head-to-head comparisons with validated prognostic models and multidisciplinary clinical assessment remain limited.

## 4 AI, health equity, and implementation challenges

AI shows promise in ovarian cancer (OC) management, but implementation requires reliable performance across populations and healthcare systems, not accuracy alone. Within a broad cancer-control framework, prevention includes early risk identification, triage, earlier diagnosis, and access to BRCA1/2/HRD testing for high-risk groups. AI should be judged by whether it reduces diagnostic delays, improves referral pathways, expands molecular stratification, and promotes equitable care.

### 4.1 Data bias and underrepresentation

Current OC AI evidence appears concentrated in high-income countries and tertiary centers, whereas studies including low- and middle-income country (LMIC) populations remain limited. This matters because OC outcomes are shaped by unequal access to diagnosis, pathology, imaging, specialist care, molecular testing, treatment, and trials (32). Bias may also arise from differences in histological subtype distribution, equipment, pathology workflows, molecular testing availability, and treatment pathways. Models trained in high-resource settings may not generalize to community hospitals or resource-limited settings without local validation. AI may address access barriers in LMICs only if adapted to local infrastructure, workforce capacity, and population needs (33).

### 4.2 Clinical translation and real-world implementation

A major challenge is the gap between retrospective performance and prospective clinical utility. Many OC AI studies report strong AUC, sensitivity, or specificity but provide limited evidence on workflow integration, clinical impact, cost-effectiveness, or patient outcomes. A meta-analysis of ultrasound-based AI reported pooled sensitivity of 81% and specificity of 92%, yet routine adoption still requires prospective validation (34). TRIPOD+AI emphasizes intended use, external validation, fairness, and clinical utility beyond predictive accuracy (35). In OC, AI should be evaluated within workflows such as adnexal mass triage, pathology review, HRD-related testing prioritization, treatment selection, referral decisions, and recurrence monitoring. DECIDE-AI, SPIRIT-AI/CONSORT-AI, and FUTURE-AI guide early clinical evaluation, prospective trials, and trustworthy implementation (36–38).

Limited prospective evidence in OC does not mean implementation is unrealistic. Although OC differs from breast cancer in prevalence, screening feasibility, and diagnostic pathways, large-scale prospective and real-world AI-supported mammography studies show that AI can reduce clinician workload while maintaining safety and performance (39, 40). These examples support prospective, workflow-integrated, and population-aware evaluation strategies for OC AI.

### 4.3 Equity-oriented AI for ovarian cancer prevention and control

Future OC AI should be designed for equitable implementation as well as accuracy. Pathology-based molecular AI, such as DeepHRD, may help prioritize patients for HRD-related testing when genomic sequencing is unavailable or delayed, but it should complement rather than replace validated genomic assays (12). Likewise, ultrasound-based AI may support risk-based triage of adnexal masses and timely referral where specialist expertise is limited (22). Equity-oriented AI also requires infrastructure-aware deployment. Digital pathology, teleconsultation-supported imaging review, and AI-assisted triage may expand expert interpretation when adapted to local resources and workflows (33). However, even H&E-based AI requires scanners, computing resources, trained personnel, data governance, and quality control. Future studies should treat inclusive datasets, local validation, affordability assessment, workforce training, and post-deployment monitoring as part of model readiness, consistent with the early validation profiles summarized in **Table 1**.

## 5 Clinical implications and future directions

Future ovarian cancer (OC) AI research should move from model development toward implementation- and outcome-oriented design. The key question is not whether AI can improve AUC, C-index, sensitivity, or specificity, but whether it improves clinically meaningful outcomes across prevention and control. These outcomes include diagnostic delay, earlier-stage detection, referral accuracy, unnecessary surgery, access to BRCA1/2/HRD testing, treatment decisions, recurrence monitoring, and patient outcomes. Future frameworks should also consider patient experience, healthcare resource utilization, and long-term effectiveness.

### 5.1 Redefining clinical utility and regulatory pathways

At present, OC AI should be regarded as an investigational or supportive tool rather than part of routine care. Before deployment, future studies should define intended use, target population, clinically relevant benchmark comparators, decision thresholds, calibration requirements, and measurable clinical utility, rather than reporting AI performance in isolation. Reporting and evaluation frameworks, including TRIPOD+AI, DECIDE-AI, SPIRIT-AI/CONSORT-AI, and FUTURE-AI, emphasize intended use, validation, fairness, prospective evaluation, and trustworthy implementation (35–38). Clinical utility should be assessed by whether AI improves referral pathways, reduces unnecessary surgery, expands molecular testing access, supports treatment selection, or improves patient outcomes.

### 5.2 Moving from retrospective validation to prospective evidence

The most urgent priority is to move from retrospective validation to prospective evidence. Future studies should evaluate discrimination, calibration, decision impact, workflow feasibility, safety, and cost-effectiveness in multicenter and real-world settings. Risk context is essential. Because OC has low prevalence in average-risk populations and false positives may cause unnecessary surgery and psychological harm, early AI evaluation may be more appropriate in risk-enriched groups, such as individuals with pathogenic BRCA1/2 variants, strong family history, or high-risk profiles. Lessons from AI-supported mammography, including nationwide real-world implementation evidence and the MASAI randomized trial, show the value of prospective and real-world evaluation, but these pathways cannot be directly transferred to OC because validated population-level screening pathways are lacking (39, 40). OC AI studies may require risk-enriched recruitment, adaptive designs, and longer follow-up to assess stage shift and survival benefit. Robust health-economic analyses should accompany clinical evaluations to inform policy and reimbursement decisions.

### 5.3 Building global data consortia and equity standards

Future OC AI should shift from an algorithm-centered paradigm to a data- and equity-centered paradigm. International consortia should curate benchmark pathology, imaging, laboratory, and molecular datasets from underrepresented regions, including low- and middle-income countries. These efforts should include privacy governance, annotation standards, local research leadership, benefit sharing, subgroup performance reporting, and ethical data use. Equity metrics, affordability, infrastructure requirements, and post-deployment monitoring should become part of evaluation. Future research should ask not only whether AI works, but whether it enables timely, affordable, and equitable OC care where it is most needed.

## 6 Conclusion

Ovarian cancer remains difficult to detect early and manage effectively because of nonspecific symptoms, biological heterogeneity, and unequal access to diagnostic and molecular resources. Over the past five years, AI has shown potential in blood-based risk stratification, ultrasound-assisted assessment, cfDNA-based detection, pathology-based molecular prediction, treatment response estimation, and prognostic modeling.

Clinical readiness varies across applications. Blood/laboratory-based AI and ultrasound-assisted adnexal mass assessment appear closest to workflow-based evaluation because they can complement existing diagnostic pathways. Pathology-based AI for HRD and treatment response prediction is transitional and should support, not replace, validated genomic testing. AI-enabled population screening, cfDNA-based early detection, and multimodal prognostic models remain preliminary because prospective validation, benchmarking, and clinical benefit evidence are limited.

For clinicians, AI should remain an adjunct decision-support tool. For trialists and policymakers, priorities include prospective benchmarked evaluation, regulatory oversight, cost-effectiveness, infrastructure readiness, and equitable deployment.
